# Pulmonary vascular resistence as prognostic factors of long-term survival in patients undergoing pulmonary endarterectomy

**DOI:** 10.1186/1749-8090-8-S1-O114

**Published:** 2013-09-11

**Authors:** J Lindner, D Ambroz, P Maruna, O Pecha, P Jansa

**Affiliations:** 1 2nd Surgical Department of Cardiovascular Surgery, First Faculty of Medicine, Charles University in Prague and General University Hospital, Prague, Czech Republic; 2 Institute of Pathological Physiology and the 3rd Department of Internal Medicine, First Faculty of Medicine, Charles University in Prague and General University Hospital, Prague, Czech Republic; 3 Technology Centre of the Academy of Sciences, Prague, Czech Republic; 4 2nd Department of Internal Medicine Department of Cardiology and Angiology, First Faculty of Medicine, Charles University in Prague and General University Hospital, Prague, Czech Republic

## Bckground

Pulmonary vascular resistance (PVR) in patients with chronic thromboembolic pulmonary hypertension (CTEPH) is well-known prognostic parameter of the disease. The aim of this study was to determine a predicting role of PVR for short- and long-term postoperative mortality in patients undergoing pulmonary endarterectomy (PEA).

## Methods

192 patients with CTEPH underwent PEA using cardio-pulmonary bypass (CPB) and deep hypothermic circulatory arrest and were included into study. Data of PVR were collected before surgery, the day of surgery and 48 h after PEA. The effect of the initial PVR, post-surgery PVR and its relative decrease on the survival rate was investigated. Kaplan-Meier method was used to estimate survival probability for three couples of the sub-samples. The equalities of the survival functions were tested using Gehan-Wilcoxon and Cox-Mantel tests for short- and long-term survival, respectively. P-values less than 0.05 were considered as statistically significant.

## Results

Overall hospital mortality was 5.2 %. Cumulative 1-year, 3-year and 5-year survival was 91,6%, 87,8% and 80,1%, respectively. No difference in short and long-term survival was found between groups with initial PVR above and below 1000 dyn.s.cm-5 (p=0.833 and p=0.808, respectively). Short-term cumulative survival was significantly higher in group with post-PVR below 250 dyn.s.cm-5 (p=0.006), but the long-term survival difference was not significant. Both short- and long-term cumulative survival was significantly higher in sub-sample with decrease of PVR more than 70% (p<0.001, and p=0.005, respectively).

## Conclusion

Long-term survival after pulmonary endarterectomy can be successfully prognoses by level of decrease of PVR after PEA. On the basis of our analysis and statistic comparison in our group of patients most relevant testing prognosting factor was reduction of PVR after operation more than 70% of preoperative level.

